# Human metapneumovirus infections on the ICU: a report of three cases

**DOI:** 10.1186/2110-5820-2-30

**Published:** 2012-07-19

**Authors:** Lenneke E M Haas, Natasja X de Rijk, Steven F T Thijsen

**Affiliations:** 1 Department of Intensive Care Medicine, Diakonessenhuis, Utrecht, The Netherlands; 2 Department of Microbiology, Diakonessenhuis, Utrecht, The Netherlands; 3 Diakonessenhuis Utrecht, PO box 80250, 3508, TG, Utrecht, the Netherlands

**Keywords:** Human metapneumovirus, hMPV, Respiratory tract infections, Intensive care, Respiratory insufficiency

## Abstract

Although human metapneumovirus (hMPV) is primarily known as a causative agent of respiratory tract infections in children, the virus also can cause respiratory infections in adults. hMPV infections tend to be mild and are self-limiting, but the infections can be severe in the elderly and immunocompromised patients. Because hMPV infection is quite common, it should be considered in every patient with respiratory failure in the intensive care unit (ICU). We describe three adult patients, including a young pregnant woman, with hMPV infection who required admission to our ICU. Two of them developed respiratory failure with indication for mechanical ventilation.

## Review

Human metapneumovirus (hMPV) was first isolated in the Netherlands in 2001 and was classified as the first human member of the *Metapneumovirus* genus in the subfamily *Pneumovirinae* of the family *Paramyxoviridae* belonging to the order *Mononegavirales*. Serologic studies of antibodies against hMPV indicate that the virus is not new and circulated in humans for at least 50 years before 2001. hMPV is genetically most similar to the Pneumoviridae family (a Paramyxoviridae subfamily), of which respiratory syncytial virus (RSV) is a prominent member. It is an enveloped, negative single-strand RNA virus. Phylogenetic analysis identified two subgroups of hMPV, subgroup A and B, and two clades within each of these subgroups [1]. Both subtypes may co-circulate simultaneously, but during an epidemic one subtype usually dominates. The virus was named human metapneumovirus because of its genetic similarity to the avian pneumovirus [2].

The virus is prevalent in all age groups, with peak incidence in pediatric and elderly patients. It is the second most common cause of lower respiratory tract infection (LRTI) in young children, after RSV [3]. Several series of children with RTI have been published, and they report highest rates among children younger than age 1 year (4–15%) [4-9]. Serologic studies show that virtually all children have been exposed to the virus by age 5 years [10,11]. Reinfections are common and appear to be a mild but can be severe. Although most reports concern children, hMPV also is an important cause of RTI in adults [12,13]. hMPV infection rates in adults appear to be lower than in children and infection often is asymptomatic, but in the elderly, the immunocompromised, and patients with underlying pulmonary and/or cardiac disease hMPV can cause severe RTI [14-18].

hMPV seems to be distributed worldwide and has a seasonal distribution comparable to that of the influenza virus; it tends to strike in the late winter and early spring [7]. Transmission is by direct or close contact with contaminated secretions, which may involve saliva, droplets, or large particle aerosols. The incubation period is approximately 3 to 6 days [19]. The signs and symptoms of hMPV infection are indistinguishable from those caused by RSV. Like RSV, hMPV has a tropism for the respiratory epithelium. The patient may be asymptomatic or symptoms may range from mild upper RTI symptoms to severe pneumonia. Most patients present with cough, dyspnea, and fever. Sputum production, wheezing, sore throat, pneumonia, bronchi(oli)tis, conjunctivitis, and otitis media are other reported symptoms [20].

A general respiratory virus culture obtained by nasal wash or nasopharyngeal swab should be performed in patients with clinical symptoms of LRTI. Virus isolation, however, is relatively difficult, because hMPV grows slowly and inefficient in cultures. Detection techniques that have been developed include identification by reverse transcriptase-polymerase chain reaction (RT-PCR) assay, enzyme-linked immunosorbent assay (ELISA), and the rapid culture technique known as shell vial amplification. The first is the most sensitive method for diagnosis of hMPV infection.

Treatment of hMPV infection is supportive. Ribavirin and polyclonal intravenous immune globulin has been shown to be effective in vitro and in mice but has not been tested sufficiently in humans yet [21,22]. Hospitalized patients with hMPV infection should be cared for in a separate room. There has been no vaccine available until now.

Although hMPV usually causes mild RTI, it can cause severe disease in vulnerable patients.

As far as we know, there are not many publications about hMPV in the adult intensive care unit (ICU). We report three cases of adult patients with hMPV infection, who required admission to our ICU. The patients were not admitted during the same period.

## Case 1

A 38-year-old man was admitted to the pulmonology ward because of fever, progressive dyspnea, and a productive cough lasting several days. He was known to have chronic obstructive pulmonary disease (COPD), diabetes mellitus, and schizophrenia, for which salbutamol, fluticasone inhalations, metformin, and clozapine was prescribed.

At physical examination, a slightly dyspneic, anxious, obese patient was seen, with a blood pressure of 140/79 mmHg, heart rate 152 beats per minute, temperature of 40°C, and oxygen saturation was 91% without supplemental oxygen. Further physical examination was not remarkable; lung auscultation revealed normal breath sounds.

Laboratory examination showed: CRP 86 mg/l (<10 mg/l), ESR 87 mm/h (1–7 mm/h), Hb 9.7 mmol/l (6.9- 10.0 mmol/l), Hct 0.46 l/l (0.38-0.49 l/l), MCV 85 fl (82–98 fl), thrombocytes 230 x10E9/l (150–350 x10E9/l), leucocytes 12.5 x10E9/l (4.0-10.0 x10E9/l), arterial blood analysis: pH 7.43 (7.35-7.45), pCO_2_ 4.9 kPa (4.7- 6.4 kPa), pO_2_ 6.6 kPa (10.0-13.3 kPa), HCO3 23.9 mmol/l (22.0-29.0 mmol/l), base excess 0.0 mmol/l (−3.0-3.0 mmol/l), oxygen saturation 99% (95-98%), lactate 1.5 mmol/l (0.5-2.2 mmol/l), amylase 68 U/l (<100 U/l), creatinine kinase 371 U/l (<175 U/l), and glucose 11,0 mmol/l (3.0-7.7 mmol/l). Kidney function and electrolytes were within the normal range and, except for a gamma-GT of 171 U/l (<50 U/l), liver enzymes were normal. Chest x-ray (CXR) showed a consolidation in the left lung.

Cultures of blood and sputum, *L. pneumophilia* and *S. pneumoniae* antigen testing in urine, testing for tuberculosis (auramine staining and enzyme-linked immunosorbent spot (ELISPOT) assay) and human immunodeficiency virus, serologic test for Legionella, Mycoplasma, and Chlamydia infection all were negative. Viral cultures of the throat showed a positive PCR for hMPV (see Appendix, Ct 32 in the throat), whereas other viruses (adenovirus, influenza A and B, parainfluenza 1, 2, and 3, rhinovirus, and RSV A and B) tested negative.

Initially he was treated with ceftriaxone, steroids, and bronchodilators (anticholinergics and beta-adrenergic receptor agonist), but because of progressive dyspnea the antibiotic regime was switched the next day to penicillin combined with erythromycin. Nevertheless, he developed respiratory failure and was admitted to our ICU for mechanical ventilation. According to our ICU protocol, the penicillin was replaced by cefotaxime, as part of the selective digestive decontamination strategy [23,24]. After 5 days, mechanical ventilation was discontinued, and 4 days later he was discharged from the hospital in good clinical condition.

## Case 2

A 73-year-old man presented to the emergency room (ER) with progressive dyspnea and a productive cough lasting 1 day, without fever. He also complained of nausea and anorexia.

He had known COPD Gold stage IV, for which he was being treated with tiotropium, salmeterol, salbutamol, fluticasone, and corticosteroids maintenance therapy (prednisone 5 mg once daily).

Physical examination showed an acutely ill cachectic man, who was tachypneic and dyspneic. He was not able to speak full sentences. Blood pressure was 189/94 mmHg, heart rate 102 beats per minute, temperature was T 36.3°C, and the saturation measured was 97% with 6 liters/minute supplemental oxygen. Auscultation of the lungs revealed bilateral wheezing.

Laboratory results showed: CRP 28 mg/l (<10 mg/l), ESR 32 mm/h (1–7 mm/h), Hb 9.3 mmol/l (6.9-10.0 mmol/l), Hct 0.45 l/l (0.38-0.49 l/l), MCV 94 fl (82–98 fl), thrombocytes 194 x10E9/l (150–350 x10E9/l), leucocytes 7.4 x10E9/l (4.0-10.0 x10E9/l), arterial blood analysis: pH 7.33 (7.35-7.45), pCO_2_ 7.7 kPa (4.7-6.4 kPa), pO_2_ 16.6 kPa (10.0-13.3 kPa), HCO3 29.6 mmol/l (22.0-29.0 mmol/l), base excess 2.1 mmol/l (−3.0-3.0 mmol/l), oxygen saturation 99% (95-98%), lactate 1.5 mmol/l (0.5-2.2 mmol/l), and glucose 7.0 mmol/l (3.0-7.7 mmol/l). Kidney function, electrolytes, and liver enzymes all were in the normal range. CXR showed severe emphysema but no other significant abnormalities.

Antibiotic therapy (amoxicillin/clavulanate in combination with a single dose of gentamicin) was started after cultures had been taken, and dexamethasone 10 mg intravenous once daily combined with bronchodilators were started. He was transferred to our ICU for noninvasive positive pressure ventilation.

Serologic testing for Chlamydia, Mycoplasma, and Legionella and also testing (both serology and PCR) for *Coxiella burnetti* were negative. Sputum culture revealed *Moraxella catarrhalis* and *S. aureus* in limited numbers. PCR of both nose and throat swabs were positive for hMPV (cycle threshold (Ct) 23 in the nose and Ct 26 in the throat). Other viruses (adenovirus, influenza A and B, parainfluenza 1, 2, and 3, rhinovirus, and RSV A and B) tested negative.

Despite this therapy, further deterioration developed, and in view of the end-stage COPD and his advance directive, treatment was restricted and he deceased soon.

## Case 3

A 24-year-old woman, with no previous medical history, was admitted to the obstetrics/gynecology ward during the 30^th^ week of an, until then, uncomplicated pregnancy (G1P0). She was suffering from fever and continuous pain in the right flank related to breaths since 3 days. Urine-analysis showed leukocyturia (242/μl, with 39 erythrocytes/μl) and bacteriuria. After cultures of urine, blood, and cervix were taken, amoxicillin/clavulanate was started for a urinary tract infection. Because of progressive tachypnea and the suspicion of progressive sepsis, antibiotic therapy was switched to a third-generation cephalosporin and subsequently to meropenem the next day because of further clinical deterioration. CXR on admission was normal. Despite therapy, respiratory insufficiency developed and she was transferred to our ICU. On admission to the ICU, she was febrile (body temperature 39°C), tachycardic (heart rate 132 beats/min), hypotensive (blood pressure 82/50 mmHg), and tachypneic (respiratory rate 40 breaths/min). Saturation without supplemental oxygen was 96%. Arterial blood gas analysis showed (normal reference ranges for pregnant woman in third trimester of pregnancy between brackets [25]): pH 7.41 (7.39-7.53), PaCO_2_ 4.4 kPa (3.3-4.4 kPa), PaO_2_ 11.7 kPa (12.2-14.2 kPa), HCO3 20.6 mmol/l (16–22 mmol/l), and saturation 97% (95-98%). Further laboratory examination showed: Hb 4.8 mmol/l (5.9-9.3 mmol/l), Ht 0.23 l/l (0.28-0.40 l/l), MVC 88 (81–99), leucocytes 13.8/nl (5.9-16.9/nl) with 12.7 neutrophils/nl (3.9-13.1 neutrophils/nl), platelets 179/nl (146-429/nl), CRP 231 mg/l (0.4-8.1 mg/l). Another CXR again showed no abnormalities. Cardiotocography was performed repeatedly and showed no abnormalities. Ultrasound of the kidneys showed dilatation of the pyelum, particularly in the right kidney with hypoechoic areas, which could be due to pyelonephritis. Drainage was performed immediately and clear urine was released. However, symptoms persisted with progressive oxygen demand (40% oxygen by Venturi mask), and a computed angiography was performed. Pulmonary embolism was ruled out, but to our surprise bilateral alveolar consolidations were seen, not visible on both CXRs performed earlier.

Both *L. pneumophilia* and *S. pneumoniae* antigen testing in urine were negative. *E. coli*, susceptible to amoxicillin, was cultured in the urine. A general respiratory virus culture was obtained by a nasopharyngeal swab, and PCR was positive for hMPV (Ct 24 in the throat and Ct 33 in the nose). Other viruses (adenovirus, influenza A and B, parainfluenza 1, 2, and 3, rhinovirus, and RSV A and B) all tested negative. Fortunately, no mechanical ventilation was necessary. She was treated with amoxicillin and high-dose oxygen, and she could be transferred to the obstetric ward after 3 days. A healthy girl was born 6 weeks later.

## Discussion

We report three adult patients with severe hMPV infection who required treatment in the ICU. Two of them developed respiratory failure with indication for mechanical ventilation and one of these eventually died, also as a consequence of his underlying pulmonary disease. Although the relevance of viral detection is always questionable, because since viral detection does not systematically mean viral infection and the virus could be a bystander rather than a true pathogen, we think that these patients really suffered from hMPV infection, because there were no indications for another causative organism.

It is generally known that hMPV infection is common among children, but it seems that hMPV also plays a significant role in adult patients. The elderly and immunocompromised patients, including pregnant women, are especially at risk, and hMPV can cause severe RTI in these patients [26-29]. Previously, most of the infections were attributed to influenza, but both RSV and hMPV are now considered to be at least equally important. RSV infections also are not solely restricted to pediatric and high-risk adult populations. Healthy adults are infected repeatedly throughout their lives with RSV and typically have symptoms restricted to the upper respiratory tract. Studies in the United States have shown that the annual incidence of RSV infection was nearly twice that of influenza A and that annually 3–7% of the healthy elderly persons and 4–10% of high-risk patients developed a RSV infection. RSV and influenza A infections resulted in comparable lengths of stay, need for intensive care monitoring, and mortality (8% and 7%, respectively) [30,31]. In data compiled by the Centers for Disease Control, RSV pneumonia is estimated to result in as many as 199,000 deaths per year globally [32]. In both elderly and immunosuppressed patients, RSV is an often unrecognized cause of LRTI [33-35]. We believe that, due to the similarities between RSV and hMPV, the latter might be of the same importance as RSV. Besides hMPV and RSV, other viruses can cause RTI, such as adenovirus, influenza, parainfluenza and, although less frequently, enterovirus. Distinguishing the viruses clinically is difficult; diagnostic testing includes culture, RT-PCR, and serology.

We analyzed all PCR tests for respiratory viruses from the past 19 months in our hospital. A total of 487 patients were tested for hMPV because of symptoms of RTI. More than half of them (283 = 58%) concerned adult patients, and 14 (4.94%) patients were positive. In the remaining 204 pediatric patients, 12 (5.88%) tested positive.

These three cases occurred in the winter: the first case in January 2010, the second in March 2011, and the last in January 2011. In 2011, at total of 277 persons were tested for hMPV. More than 70% (198/277 = 71.5%) concerned adult patients and four of them (4%) tested positive. During the period from January to March 2011, 112 patients were tested, including 77 adults; 3 of them (3.9%) tested positive. In this series, the incidence of hMPV is lower in the children group (approximately 2% versus approximately 4%), but this can be caused by a selection bias, because only samples from children with a negative RSV antigen test were analyzed further by PCR testing. These incidences are lower than the incidence reported by Li and colleagues [13]. We think that the incidences that we found are a considerable underestimation. The real incidence of hMPV infections is difficult to measure or estimate; first, because a great part of the patients with respiratory symptoms presenting to our hospital are not tested for viral infections. Second, a great part of the hMPV infections is asymptomatic or mild, and these patients do not present to the hospital at all, so the asymptomatic and less severe cases also are missed.

The first patient was known with diabetes mellitus and used inhalation steroids. Therefore, he could be considered immunocompromised and the underlying pulmonary disease made him vulnerable for progressive respiratory failure due to a viral infection. Because no microorganism was cultured, we considered a viral cause and PCR for respiratory viruses was performed. Although sputum cultures can be negative, even in the case of true bacterial pneumonia, and the urine tests for *Legionella pneumophilia* detects only antigens of serotype 1 and also has no 100% sensitivity, we think that the most plausible diagnosis in this case is a severe hMPV infection.

The second patient was known with serious COPD and used both inhalation steroids and oral corticosteroids maintenance therapy; therefore, he also could be considered immunocompromised. The hMPV infection in combination with his explicit wishes not to use invasive mechanical ventilation, finally resulted in his death, although it cannot be ruled out that a bacterial infection with the cultured *Moraxella catarrhalis* and *S. aureus* might have played a role.

Although presentation with classic symptoms of fever and dyspnea, the diagnostic process was complicated in the last patient. Because she had no respiratory symptoms and pulmonary auscultation and CXR were not remarkable, we focused on the leukocyturia and bacteriuria in combination with the flank pain and bilateral hydronephrosis. It was not before we got the viral diagnostic test results and saw the consolidations on CT images that we reconsidered pneumonia as the most probable diagnosis. Retrospectively, we can say that the bacteriuria and pyelumdilatation has misled us, leading to unnecessary drainage of both kidneys. Bilateral hydronephrosis can occur during pregnancy due to enlargement of the uterus. Sometimes it can be difficult to distinguish clinically between pneumonia and urosepsis as the cause of the respiratory deterioration. Maybe the flank pain was caused by the pneumonia.

We think the hMPV was the causative pathogen, but a bacterial pneumonia could not completely be ruled out, because the patient was treated with broad-spectrum antibiotic without sampling of the lower respiratory tract. An acute lung injury due to urinary sepsis could be another possible explanation.

It is generally known that the immunocompromised patient is susceptible for hMPV infection. Even though pregnant women can be considered mildly immunodeficient, severe infection in pregnant women has not been, as far as we know, reported previously. The unborn child was unaffected by the virus and was born healthy.

## Conclusions

hMPV can cause serious RTI in vulnerable adult patients. Because hMPV infection is quite common, it should be considered in every patient with respiratory failure in the ICU.

## Appendix

● RT- PCR

After addition of 10 μl of internal control (Phocine Distemper Virus), viral RNA or DNA was isolated from 190 μl of nasopharyngeal swab supernatant by use of the Total Nucleic Acid High Performance Kit (protocol Total NA HP200) on the MagNA Pure LC (Roche Diagnostics). cDNA synthesis and amplification were performed with 10 μl mastermix (TaqMan(R) Fast Virus 1-step Master Mix (#4444436, Life Technologies/Applied Biosystems) and 10 μl eluate on an ABI 7500 SDS. Specific primers and probes were used for the detection of HMPV [16], RSV types A and B, rhinovirus, PIV-3 [17], PIV1-2, adenovirus [18], and influenza A and B [19]. Amplified products were detected using pathogen-specific FAM-, VIC-, or CY5-labeled Taq-Man probes. Positive and negative controls were included in each run.

## Competing interests

The authors declare that they have no competing interests.

## Authors’ contributions

The three authors have each contributed to the writing of the manuscript. LH wrote the initial review, and NdR wrote the case series. Subsequently, LH edited the case series and NdR edited the review. ST corrected the initial manuscript and wrote additional paragraphs. All authors read and approved the final manuscript.
